# The molecular pathogenesis of craniopharyngiomas

**DOI:** 10.20945/2359-3997000000600

**Published:** 2023-02-07

**Authors:** Marina Lanciotti Campanini, João Paulo Almeida, Clarissa Silva Martins, Margaret de Castro

**Affiliations:** 1 Universidade de São Paulo Faculdade de Medicina de Ribeirão Preto Departamento de Clínica Médica Ribeirão Preto SP Brasil Departamento de Clínica Médica, Faculdade de Medicina de Ribeirão Preto, Universidade de São Paulo, Ribeirão Preto, SP, Brasil; 2 Mayo Clinic Department of Neurosurgery Jacksonville FL United States Department of Neurosurgery, Mayo Clinic, Jacksonville, FL, United States; 3 Universidade Federal do Mato Grosso do Sul Faculdade de Medicina Campo Grande RS Brasil Faculdade de Medicina, Universidade Federal do Mato Grosso do Sul, Campo Grande, RS, Brasil

**Keywords:** Craniopharyngioma, adamantinomatous, papillary, *CTNNB1*, *BRAF V600E*, molecular pathogenesis

## Abstract

Research from the last 20 years has provided important insights into the molecular pathogenesis of craniopharyngiomas (CPs). Besides the well-known clinical and histological differences between the subtypes of CPs, adamantinomatous (ACP) and papillary (PCP) craniopharyngiomas, other molecular differences have been identified, further elucidating pathways related to the origin and development of such tumors. The present minireview assesses current knowledge on embryogenesis and the genetic, epigenetic, transcriptomic, and signaling pathways involved in the ACP and PCP subtypes, revealing the similarities and differences in their profiles. ACP and PCP subtypes can be identified by the presence of mutations in *CTNNB1* and *BRAF* genes, with prevalence around 60% and 90%, respectively. Therefore, β-catenin accumulates in the nucleus-cytoplasm of cell clusters in ACPs and, in PCPs, cell immunostaining with specific antibody against the V600E-mutated protein can be seen. Distinct patterns of DNA methylation further differentiate ACPs and PCPs. In addition, research on genetic and epigenetic changes and tumor microenvironment specificities have further clarified the development and progression of the disease. No relevant transcriptional differences in ACPs have emerged between children and adults. In conclusion, ACPs and PCPs present diverse genetic signatures and each subtype is associated with specific signaling pathways. A better understanding of the pathways related to the growth of such tumors is paramount for the development of novel targeted therapeutic agents.

## INTRODUCTION

Craniopharyngiomas (CPs) are intracranial neoplasms located mainly in the sellar and supra-sellar regions, along the anatomical developmental pathway of the craniopharyngeal duct. CPs’ incidence is around 0.16-2 cases per million persons per year ( Table 1 ), accounting for 2% to 5% of all primary intracranial neoplasms and for 5.6% to 13% of intracranial tumors in children ( 1 - 6 ). The majority of studies show no sex asymmetry; however, more recently, Feng and cols. (2019) observed that men were more commonly affected than women were, specifically in a sample of patients of Chinese origin ( 2 , 5 - 7 ).

**Table 1:** Classification of craniopharyngiomas

	Adamantinomatous	Papillary
Incidence	0.16–2 cases per million persons/ year	
Frequence (%)	90%	10%
Age (years)	5–14 and 55–74	40–53
Clinical presentation	Predominant inicial symptoms: increased intracranial pressure; visual field defects; hypopituitarism; cognitive impairment; overweight	
Macroscopy	Multicystic tumor (dark motor-oil’ content) with or without solid components	Purely or predominantly solid (if cystic: viscous yellow content)
Microscopy	Multicystic, “stellate reticulum”, “wet keratin", occasional calcification, finger-like protrusions into brain bordered by palisading cells, chronic inflammation in peritumoral brain	Papillary growth pattern with mature nonkeratinizing squamous epithelium; no wet keratin; no calcification; well circumscribed neoplastic epithelium, adjacent brain tissue infiltration usually absent
Molecular pathogenesis markers	*CTNNB1* mutation	*BRAF V600E* mutation

CPs were first described in 1857 by Friedrich Albert von Zenker. In 1904, Jakob Ederheim described CPs’ histopathological characteristics, suggesting that these tumors arose from ectodermal embryonic remnants of the primitive mouth or stomodeum ( 8 , 9 ). In 1932, Harvey Cushing described his experience with the management of CPs and characterized those as “the most baffling problem which confronts the Neurosurgeon” ( 10 , 11 ).

CPs are classified as histologically benign grade I tumors by the World Health Organization (WHO) ( 12 ). However, CPs are challenging tumors to treat due to their location and close relationship to neurovascular structures, including the optic apparatus, third ventricle and hypothalamus, pituitary stalk, and internal carotid artery and its branches, which may preclude gross surgical removal to avoid new postoperative neurological deficits ( 13 ). Subtotal resection of CPs is associated with higher recurrence rates and adjuvant radiation treatment is usually recommended ( 13 ). Due to their location and relationship with important brain structures, their pattern of recurrence and need for multimodality treatment, CPs are often associated with significant morbidity, including hypopituitarism, hypothalamic impairment, and visual, neurological, and cognitive deficits. In addition to these disabling complications, obesity due to hypothalamic disorders has been highlighted as a critical adverse complication in patients with CPs ( 10 , 14 , 15 ). The exact mechanisms responsible for the development of hypothalamic obesity are not yet fully understood. As the hypothalamus integrates peripheral neural and hormonal afferent signals of satiety and energy reserve and acts directly on efferent signals that affect energy supply and expenditure, damage to the hypothalamic control system can result in weight gain, as demonstrated by the presence of obesity or overweight in 51.4% of the patients at diagnosis, which increased to 86.5% after surgical treatment ( 16 - 19 ). In view of these serious chronic morbidities and increased mortality during long-term follow-up, CPs have been associated with the lowest quality of life (QoL) among the different types of pediatric brain tumors ( 20 - 23 ). The impact on QoL has also been observed in adults presenting CPs ( 24 ).

Two theories have been considered regarding the genesis of CPs. The first theory suggests that CPs result from metaplasia of adenohypophyseal cells in the pituitary stalk or gland. The basis for this theory is CPs’ putative origin in squamous cell nests with intracellular tonofilaments, desmosome-associated tonofilaments, and kerato-hyaline granules, which could be evidence of squamous differentiation. In addition, the histochemical analyses also indicate keratinization in CPs’ epithelial cells ( 25 - 27 ). The second theory postulates that CPs arise from pituitary progenitor or stem cells representing embryonal remnants of Rathke's pouch epithelium ( 28 - 33 ). Studies in mouse models have provided insights into the importance of stem cells in the tumorigenesis of craniopharyngiomas ( 34 ).

CPs are currently divided into two main subtypes, adamantinomatous (ACP) and papillary (PCP). The subtypes share a few similarities, such as anatomic location, adult pituitary stem cell markers, glial reaction proteins, and cytokeratin expression. However, ACP and PCP present distinct morphological and histological features as well as different epidemiological and biological behavior ( 10 , 25 , 35 ). In addition, recent studies have demonstrated the differentiation of ACP and PCP subtypes according to epigenetic and molecular profiles, indicating that these tumors represent different entities ( 34 , 36 - 43 ).

The purpose of this minireview is to assess the current state of knowledge on embryogenesis and the genetic, epigenetic, transcriptomic and signaling pathways involved in the ACP and PCP subtypes, thereby revealing the similarities and differences in their profiles.

### Papillary craniopharyngioma (PCP)

PCPs have been almost exclusively described in adults, aging 40 to 53 years ( Table 1 ) with a mean age of 44.7 years ( 5 , 44 ). They often present as large tumors located in the suprasellar area and within the third ventricle ( 45 ).

Macroscopically, PCPs are mostly solid mass or mixed mass with viscous yellow cysts and solid components, but calcifications are rare ( 4 , 46 ). These tumors are well-circumscribed neoplastic epithelium, and adjacent brain tissue infiltration is usually absent ( 47 ). At the microscopic level, PCPs’ histological features resemble those of the oropharyngeal mucosa, but PCPs do not express enamel proteins, amelogenin, or enamel proteinase, as observed in ACPs, suggesting a different origin of these CP subtypes ( 4 , 46 ). PCPs are composed of mature squamous-epithelium-forming pseudopapillae and an anastomosing fibrovascular stroma with thin capillary blood vessels and scattered immune cells including macrophages and neutrophils ( 48 ). There are no peripheral palisading or stellate reticulum cells, with no wet keratin and, only occasionally, small collagenous whorls ( 42 , 47 ). PCPs are often difficult to distinguish from other suprasellar and infundibulotuberal masses, such as non-neoplastic Rathke's cleft cysts ( 42 ).

**Figure 1:**
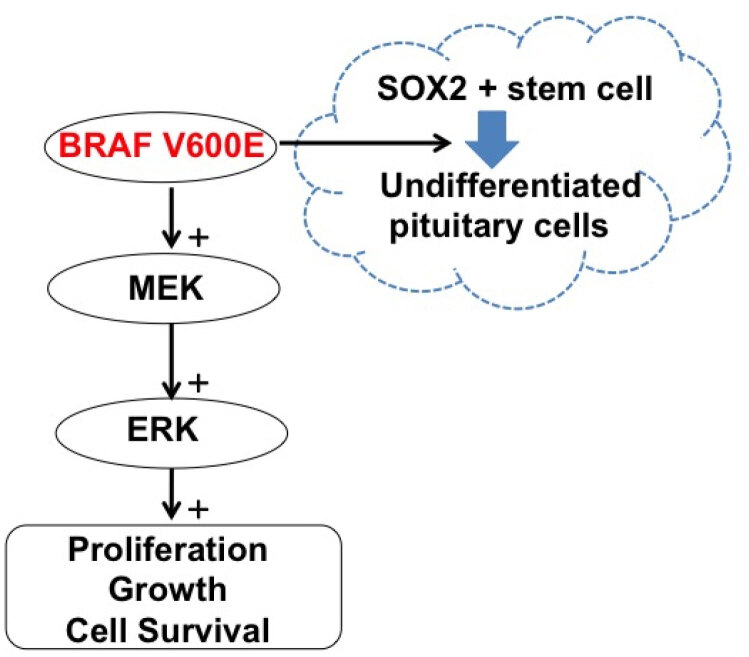
Schematic representation of the main molecular events underlying the tumorigenesis of papillary craniopharyngiomas (PCPs).

Understanding of the genetic and epigenetic profile of PCPs has significantly evolved in the last 10 years. Brastianos and cols. (2014) identified the *BRAF V600E* mutation via exome sequencing in three human PCP samples ( 41 ). The same mutation was subsequently observed in 36 out of 39 PCPs ( 48 ). *BRAF* participates in the signaling cascade of mitogen-dependent kinases (MAPK/ERK). It is a well-established oncogene that has been shown to constitutively activate serine-threonine kinase through ligands such as epidermal growth factor (EGF), platelet-derived growth factor (PDGF), and fibroblast growth factor (FGF) ( 49 ). The increased MAPK/ERK signaling leads to increased proliferative capacity of the *SOX2* ^+^ cells, preventing the pituitary from differentiating into hormone-producing pituitary cells and resulting in cell transformation and tumorigenesis, as schematically represented in Figure 1 ( 41 , 43 , 49 , 50 ).

The *BRAF V600E* mutation constitutively activates the signaling cascade of mitogen-dependent kinases (MAPK/ERK, also known as the Ras-Raf-MEK-ERK pathway). This increases the proliferative capacity of the *SOX2* ^+^ cells, preventing the pituitary's differentiation into hormone-producing pituitary cells and resulting in cell transformation and tumorigenesis.

To assess the role of the MAPK/ERK pathway during the development of PCPs, Haston and cols. (2017) crossed the *Hesx1^Cre^* ^−/+^ mice with animals *Braf* ^*V600E* /+^ or *Kras* ^G12D/+^. Genotyping the postnatal mice from birth to 3 weeks, these authors failed to identify any viable animals ( 49 ). Histological examination at 18.5 days post conception (dpc) revealed the presence of changes in the airways in both *Braf* ^V600E/+^ or *Kras* ^*G12D* /+^ mouse models, suggesting that abnormal lung development was the cause of the observed perinatal deaths. Anterior pituitary hyperplasia was observed at 12.5 dpc and was pronounced at 14.5 dpc. At 18.5 dpc, a penetrating phenotype of severe anterior pituitary hyperplasia with branched cleft was observed in all analyzed embryos. In addition, MAPK/ERK pathway expression was temporarily upregulated at 10.5 to 18.5 dpc. Moreover, most cells that presented upregulation of the MAPK/ERK pathway were located in the cleft epithelium, an area enriched by undifferentiated *Sox2* ^+^ embryonic precursors as well as by other stem cells. Therefore, the expression of the *Braf* ^*V600E*^ mutation in the mice pituitary developing seems to lead to the expansion of *Sox2* ^+^ stem cells.

The presence of *BRAF V600E* mutation and the expression of BRAFV600E protein were confirmed in five samples of human PCP. The staining of pERK1/2 was more restricted and focused on the areas around the fibrovascular nuclei. Double immunostaining revealed that the components of the squamous epithelial tumor also significantly expressed *SOX2* . The analyzes showed that 16% of these *SOX2* ^+^ cells expressed Ki67, suggesting that the proliferation of *SOX2* ^+^ cells may be responsible for the growth of PCPs ( 49 ).

*BRAF V600E* mutation, which prevalence ranges from 81%-100%, occurs almost exclusively in PCPs ( 41 , 43 ). In this way, a hallmark of the PCPs is the positive staining for a *BRAF V600E* mutation-specific antibody (VE1) while β-catenin has been located on cell membranes in PCPs ( 42 ). As the *BRAF V600E* mutation has also been described in melanomas and the treatment with MEK and BRAF inhibitors has drastically changed the evolution of that disease, MEK and BRAF inhibitors have also been considered for treatment of selected PCPs. Indeed, the first report of MEK/BRAF inhibitors for treatment of refractory PCP described a tumor reduction of 85% and 81% of the solid and cystic parts of the tumor, respectively. These findings were subsequently confirmed ( 42 , 48 ). More recently, additional studies have further reported the results of target therapy for PCPs harboring *BRAF V600E* mutations. In a series of 6 patients treated with dabrafenib, trametinib and vemurafenib, isolated or in association, there was 80% to 91% regression of the cystic and solid parts in all PCP cases, which allowed surgical and/or radiotherapy after initial medical treatment ( 51 ). Currently, phase 2 of an ongoing multicentric study is assessing the role of adjuvant MEK/BRAF inhibitors in the treatment of *BRAF V600E* -mutation-positive PCPs (NCT03224767).

The role of inflammatory pathways has also been a topic of the study of PCPs and ACPs. Liu and cols. (2016) observed that PCPs and squamous cells from ACPs have hyperexpression of triggering receptors expressed on myeloid cells-1 (TREM-1), suggesting this as a potential marker of squamous metaplasia via inflammatory pathways ( 52 ). Chen and cols. (2018) identified a dense neutrophilic inflammation in PCPs but rarely in ACPs. In fact, neutrophils may be correlated with antitumor immunity ( 53 , 54 ). Interestingly, PCPs treated with BRAF/MEK inhibitors developed a prominent inflammatory infiltrate that has been associated with significant radiologic reduction in tumor volume ( 48 , 54 ).

### Adamantinomatous craniopharyngioma

ACPs represent 90% of all craniopharyngiomas and can occur at all ages but show a bimodal distribution with peaks from 5 to 14 years old and from 55 to 74 years old ( Table 1 ) ( 6 , 55 ). The median age at ACP diagnosis in children younger than 15 years old is 8.8 ( 10 , 14 ). Hölsken and cols. (2016) demonstrated that pediatric and adult ACPs seem to have no differences either in the epigenomic or in transcriptional and methylation levels ( 43 ). These data were recently confirmed by Prince and cols. (2020), who analyzed potential age-related transcriptional differences of ACPs and found no relevant distinction between pediatric and adult ACPs ( 56 ).

Müller and cols. (2019) described the rule of 90%, whereby ∼90% of tumors are predominantly cystic, ∼90% show typically prominent calcifications, and ∼90% take up contrast media in the cyst walls, which may contain dark, greenish-brown, turbid, cholesterol-rich liquid resembling “motor oil” ( 21 , 57 ). The margins of ACPs are sharp and irregular, composed of a palisaded basal layer of cells, that may infiltrate finger-like structures which can contain whorl-like cell clusters surrounded by an intense gliosis (an inflammatory reaction in the adjacent brain), often making identification of the surgical planes difficult ( 58 ). The heterogeneous tumor epithelium is adjacent to a layer of stellate cells (known as reticulum stellate) and nodules of wet keratin formed by nuclear squamous cells or ghost cells, frequently associated with a regressive change such as cholesterol clefts with a foreign-body giant cell reaction, as are calcification and hemosiderin deposits due to chronic hemorrhage. These traits are specific features of ACPs ( 10 ). The ACP reticulum stellate is similar to the one inside the enamel organ of an embryological tooth, as in odontogenic tumors, such as adamantinoma of the mandible ( 58 ). The expression of enamel proteins and LEF1 in ACPs suggests not only their morphological, but also their functional similarities with odontogenic epithelium ( 37 , 59 ).

The pathogenesis of ACPs has been characterized by activating somatic β-catenin 1 gene ( *CTNNB1* ) mutations. Under normal conditions, the Wnt pathway regulates essential physiological processes including growth, reproduction, metabolism, and stress response, and a low level of β-catenin expression is limited to the cell membrane ( 60 ). In addition, β-catenin is also part of the adherent complex preserving cytoskeletal architecture that includes E-cadherin protein ( 61 , 62 ). Activating somatic *CTNNB1* mutations were first identified by Sekine and cols. (2002), with a prevalence ranging from 16% to 100% in the ACPs analyzed ( 37 , 38 , 43 , 63 ). This variation may be secondary to the use of variable sequencing approaches such as Sanger or next generation sequencing (NGS) and/or due to the low proportion of tumor tissue within these samples ( 21 , 42 , 64 , 65 ).

**Figure 2:**
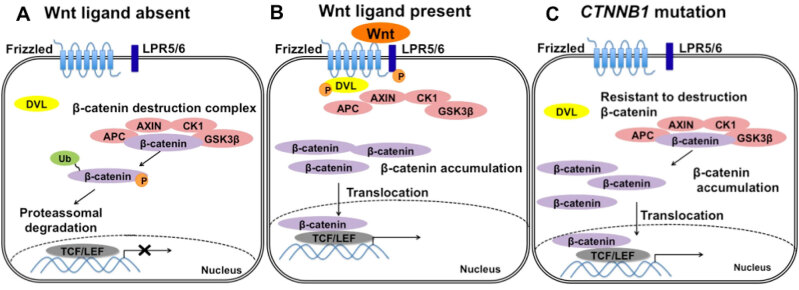
Schematic representation of the canonical Wnt/β-catenin-signaling pathway. **(A)** In the absence of a Wnt ligand, β-catenin binds to the destruction complex (APC, AXIN, CK1 and GSK3β) and is phosphorylated by CK1 and GSK3β, then ubiquitinated and degraded by the proteasome, preventing the transcription of β-catenin target genes. **(B)** In the presence of a Wnt ligand, the ligand binds to its cellular receptors (Frizzled and LRP5/6), resulting in the recruitment of DVL to the membrane, which inactivates the β-catenin destruction complex, leading to accumulation of β-catenin. β-catenin translocates into the nucleus and activates target gene transcription by interacting with *TCF/LEF* transcription factors. **(C)** In the presence of *CTNNB1* exon 3 mutation, β-catenin becomes resistant to degradation and accumulates, activating the Wnt pathway even in the absence of a Wnt ligand.

**Figure 3:**
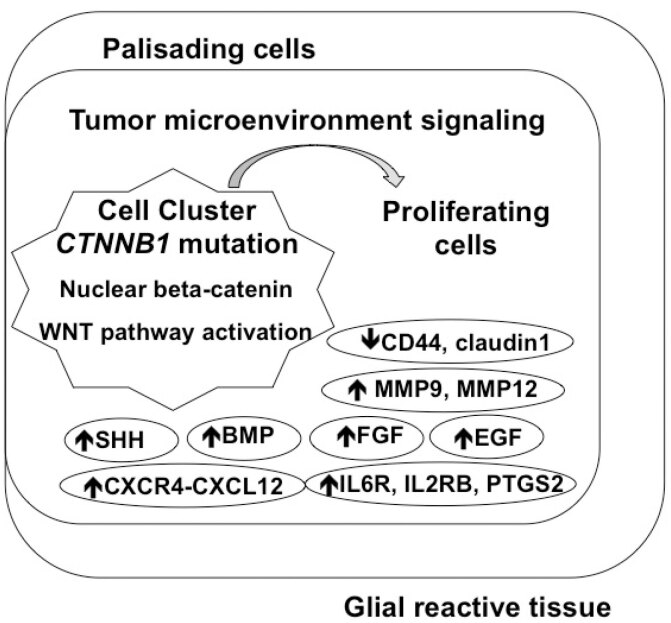
Schematic representation of the cellular compartments of the adamantonomatous craniopharyngioma (ACP) and the tumor microenvironment. The ACPs are composed of β-catenin positive cell clusters, adjacent to stellate cells known as reticulum stellate, surrounded by a palisaded basal layer of cells and intense gliosis and inflammatory reaction in the adjacent brain. These clusters could act as a paracrine tumor-signaling center by activating cells with a secretory phenotype, which may then secrete growth factors, cytokines, chemokines, and proteases, thereby changing the tumor microenvironment. Besides Wnt-pathway activation driven by the *CTNNB1* mutations, different pathways and proteins have been shown to be overexpressed in ACPs as Sonic Hedgehog (SHH), epidermal growth factor (EGF), fibroblast growth factor (FGF), bone morphogenetic protein (BMP), matrix-metallopeptidases (MMP), pro-inflammatory factors as interleukins and chemokines and their receptors (IL6R, IL2RB, PTGS2, CKCR4, CXCL12), while adhesion molecules seem to be underexpressed (CD44, claudin-1).

*CTNNB1* mutations observed in ACPs affect the exon 3, which encodes the degradation targeting box of β-catenin protein, driving its instability and aberrant nucleo-cytoplasmic accumulation that occurs in almost 96% of ACPs ( 43 ). β-catenin nucleo-cytoplasmic accumulation leads to overactivation of the Wnt pathway, which is involved in control of cellular proliferation and pituitary embryogenesis, as evidenced by the expression of downstream pathway targets such as *AXIN2, LEF1* , and *BMP4* ( Figure 2 ) ( 39 , 40 , 66 , 67 ). Interestingly, in ACPs, the nucleo-cytoplasmic β-catenin accumulation is found only in small clusters of cells, with epithelial whorl-like structures, or in a few cells near the infiltrating edge of the tumor. These areas have shown to be critical signaling centers for determining the proliferation and differentiation of ACP cells through the paracrine effect of secreted factors ( Figure 3 ) ( 40 , 68 ). This aberrant pattern of β-catenin distribution has been found in several other tumors of epithelial origin, such as tumors containing nodules of wet keratin, as pilomatricoma and calcifying odontogenic cysts ( 69 ). However, hyperactivation of the Wnt pathway and aberrant nucleo-cytoplasmic β-catenin cell clusters are hallmarks of human ACPs and such characteristics are not observed in other sellar region tumors, including the PCPs ( 37 , 43 , 48 ). Similarly, *CTNNB1* mutations appear to also be specific to ACPs and do not occur in other types of pituitary tumors or in PCPs ( 41 , 43 , 70 ). However, the coexistence of *CTNNB1* and *BRAF* mutations has been described in a small number of ACPs presenting with mixed adamantinomatous and papillary histologic features ( 46 , 65 ). In addition to pathogenesis, activation of the Wnt pathway also appears to have a prognostic role in ACPs. A higher aberrant nucleo-cytoplasmic β-catenin ratio has been associated with more aggressive disease, and *CTNNB1* mutations have also been related to worse overall survival rates ( 71 - 73 ). Apps and cols. (2018) identified the activation of the MAPK/ERK pathway in compartments of ACPs. The expression of several ligands, such as FGFs, epidermal growth factor (EGF), platelet-derived growth factor (PDGF), and ERK1/2, were co-localized with the proliferation marker Ki67 within the palisading epithelium around the clusters and neighboring reactive tissue ( 74 ). *Ex vivo* culture experiments using small pieces of human ACPs grown with and without trametinib, an inhibitor of the MAPK/ERK pathway, revealed a reduction in the immunofluorescence of pERK1/2 in ACPs treated with trametinib compared to vehicle-treated controls that was associated with a dose-dependent increase on apoptosis and significant reduction proliferation, suggesting the downstream activation of the MAPK/ERK pathway ( 74 ).

As observed with Wnt activation, strong Sonig Hedgehog (SHH) pathway activation has also been observed, not only in cell clusters, but also in the basal layer of cells in palisades, which present cells with positive Ki67 staining ( 39 , 75 ). The SHH signaling pathway has been related to pituitary embryogenesis and seems to be involved in the maintenance of tumor stem cells. In the face of this evidence, researchers have hypothesized that the SHH pathway promotes tumor growth, infiltration, and angiogenesis by the activation of transcription factors in palisade cells by autocrine and paracrine actions ( 74 - 77 ). However, Carreno and cols. (2019), paradoxically, showed that SHH pathway inhibition in human ACPs led to a significant increase in tumor cell proliferation ( 78 ).

Many studies in ACPs have demonstrated, besides Wnt and SHH pathways, a pattern of expression of fibroblast growth factor (FGF), bone morphogenetic proteins (BMPs), and transforming growth factor β (TGFβ) families ( 40 , 74 ). Activation of the epidermal growth factor receptor (EGFR) pathway also led to β-catenin stabilization in tumor cell clusters co-expressing fascin, a member of the actin cross-linking family of proteins, which has been associated with matrix adhesion, cell migration, invasion by filopodia formation, and reorganization of the actin cytoskeleton. These findings have been demonstrated in a variety of tumors, including ACPs ( 77 , 79 - 81 ). Furthermore, fascin was associated with invasive growth behavior and consequently an unfavorable prognosis for ACP patients ( 80 , 82 ).

Cytokeratin markers (KL-1, CK5/6, CK7, CK19), which predominate along the edges of the palisade cells of ACPs, are related to invasive growth behavior ( 83 ). Of note, CK8 and CK18 were increased in positive β-catenin cell clusters, as observed in squamous carcinomas, and are indicators of loss of differentiation and tumor progression. In addition, claudin-1 (CLDN1), a component of tight junctions, has been downregulated in positive β-catenin cell clusters, in finger-like protrusions, and in tumoral cells bordering brain tissue, suggesting a role in the invasiveness pattern of ACPs. Furthermore, claudins can also influence the morphology of ACPs through the formation of cysts caused by the accumulation of fluid through leakage of the endothelium, since they have an important role in cell polarity and cell-cell adhesion ( 84 ). Claudins can also increase the typical inflammation observed in ACPs and can induce morphological changes in the glial cells (Rosenthal fibers) ( 79 , 84 ). These changes are typically present in the peritumoral brain area around the finger-like protrusions and are characterized by overexpression of Tenascin-C (TN-C), nestin, vimentin, microtubule associated protein 2 (MAP2), and glial fibrillary acid protein (GFAP). In the glial reactive tissue, the presence of cholesterol crystals leads to secretion of interleukin-1B (IL-1B), which in turn acts on the local immune effector cells to drive an inflammatory response ( 74 , 85 ).

### Tumorigenic Microenvironment in ACP

In an elegant study, Gaston-Massuet and cols. (2011) developed a mutant mouse model resistant to β-catenin degradation in immature Rathke's pouch progenitor cells and provided evidence suggesting that the development of ACP is related to β-catenin activation in pituitary progenitor stem cells expressing *SOX2, SOX9* , and *p27KIP* ( 39 ). Using laser capture microdissection to assess gene expression within the different cellular compartments of the ACPs, the authors observed the enrichment of Wnt signaling expression in palisade epithelium clusters, not glial reactive tissue. In addition, the researchers observed that these progenitor cells accumulated β-catenin and formed clusters and that surviving mice developed tumors similar to human ACPs. It is important to mention, however, that Ki67 staining was not present within the positive β-catenin cluster, suggesting that the mutated β-catenin cells may not be directly responsible for ACPs’ proliferation and growth ( 39 , 76 ).

Another mouse model characterized by overexpression of mutated β-catenin in stem cells ( *Sox2* positive cells) of the adult pituitary gland also resulted in the appearance of tumors similar to human ACPs ( 86 ). It was observed a process of transient cell proliferation, the interruption of cell division and formation of clusters, which secrete (in a paracrine way) signals to neighboring cells that induce the transformation and growth of the surrounding cells, derived neither from the stem cell nor from positive β-catenin cell clusters. These clusters, therefore, may function as a tumor signaling center, leading to the hypothesis that stem cells could have a paracrine role in tumorigenesis. These cells would activate cells with a secretory phenotype associated with tumor senescence, which includes secretion of growth factors, cytokines, chemokines, proteases, and components of the extracellular compartments ( 79 ). Thus, senescent cell clusters activate a secretory phenotype that results in changes in the cellular microenvironment, characterized by inflammatory changes and immune response, which have a critical role in the pathogenesis of ACPs ( 74 , 82 , 87 ).

Inflammation seems to be closely correlated with the development of CPs. Pro-inflammatory mediators such as some interleukins (IL), including IL-6, IL-8, tumor necrosis factor (TNF), and other CXC and CC chemokines, interact with positive β-catenin cluster and their surrounding cells ( 40 , 74 , 82 ). CXCL12 and CXCR4 expressions correlate with the risk of recurrence and poor survival after resection in pediatric ACPs. Metalloproteinase-9 (MMP-9), collagen Col IV, and vascular endothelial growth factor (VEGF) may also be specific biomarkers related to the ACPs’ recurrence ( 88 , 89 ). Donson and cols. (2017) also found high levels of cytokines and chemokines, especially IL-6, CXCL1, IL-8, IL-10 and their receptors, in ACP cyst fluid and tumor tissue ( 90 ).

MMPs, regulated by β-catenin/TCF by VEGFs, are hyperexpressed in stromal capillaries and in the epithelial components of both ACPs and PCPs ( 91 , 92 ). MMPs are capable of inducing the expression of the anti-apoptotic protein B-cell leukemia/lymphoma 2 (Bcl-2) that participates in the regulation of tumor cell growth and, therefore, promotes CP growth and recurrence in an autocrine-paracrine manner ( 89 , 91 ). Using a monoclonal antibody that binds to VEGF with IL-6 receptor antagonist, Grob and cols. (2019) observed a significant cyst regression in pediatric ACPs ( 93 ). Additionally, therapy with anti-IL6 (tocilizumab) is currently being investigated in ACPs ( 94 ). The hyperexpression of IL-10 and IDO-1, immunosuppressive factors, has also been implicated as part of the pathological inflammatory microenvironment of CPs ( 90 ). In the face of all this evidence, Martinez-Barbera (2015) stated that ACPs may be an inflammation-driven tumor ( 68 ).

In conclusion, CPs can be subdivided into two groups: PCPs and ACPs. These groups differ in age distribution, clinical course, location and degree of attachment to surrounding neurovascular structures, histomorphology, and developmental pathways. The majority of ACPs and PCPs harbor single exclusive *CTNNB1* or *BRAF V600E* clonal driver mutations, indicating a different molecular origin. Furthermore, target genes from Wnt ( *LEF1* and *AXIN2* ) and SHH ( *GLI2, PTCH1* and *SHH* ) signaling pathways were up-regulated in ACPs, supporting the hypothesis that both variants of CP are different molecular entities. Additionally, protein expression and methylation profiles differ between subtypes, with hyperexpression of stem cell markers (CD133, TN-C, and MAP2) and down-regulation of CD44 and CLDN1 in ACPs, but not in PCPs. However, PCPs and ACPs also share some similarities, such as the profile of adult pituitary stem cell markers such as *SOX2, OCT4* , *KLF4* , and *SOX9* , the expression of cytokeratins, and the expression of glial reaction proteins such as GFAP, nestin, and vimentin. Recent molecular studies of CPs have unraveled patterns of biological behavior to improve the current therapies and patients’ quality of life. Further data on genetic and epigenetic targets are still needed to better illuminate the pathways involved in the development and progression of CPs and to advance the development of additional therapeutic modalities.
